# Facilitation of Male Sexual Behavior in Syrian Hamsters by the Combined Action of Dihydrotestosterone and Testosterone

**DOI:** 10.1371/journal.pone.0012749

**Published:** 2010-09-14

**Authors:** David J. Piekarski, Ned J. Place, Irving Zucker

**Affiliations:** 1 Department of Psychology, University of California, Berkeley, California, United States of America; 2 Department of Integrative Biology, University of California, Berkeley, California, United States of America; 3 Department of Population Medicine & Diagnostic Sciences, College of Veterinary Medicine, Cornell University, Ithaca, New York, United States of America; Pennsylvania State University, United States of America

## Abstract

**Background:**

Testosterone (T) controls male Syrian hamster sexual behavior, however, neither of T's primary metabolites, dihydrotestosterone (DHT) and estradiol (E_2_), even in highly supraphysiological doses, fully restores sexual behavior in castrated hamsters. DHT and T apparently interact with androgen receptors differentially to control male sexual behavior (MSB), but whether these two hormones act synergistically or antagonistically to control MSB has received scant experimental attention and is addressed in the present study.

**Methodology/Principal Findings:**

Sexually experienced male Syrian hamsters were gonadectomized and monitored 5 weeks later to confirm elimination of the ejaculatory reflex (week 0), at which time they received subcutaneous DHT-filled or empty capsules that remained *in situ* for the duration of the experiment. Daily injections of a physiological dose of 25 µg T or vehicle commenced two weeks after capsule implantation. MSB was tested 2, 4 and 5 weeks after T treatment began. DHT capsules were no more effective than control treatment for long-term restoration of ejaculation. Combined DHT + T treatment, however, restored the ejaculatory reflex more effectively than T alone, as evidenced by more rapid recovery of ejaculatory behavior, shorter ejaculation latencies, and a greater number of ejaculations in 30 minute tests.

**Conclusions/Significance:**

DHT and T administered together restored sexual behavior to pre-castration levels more rapidly than did T alone, whereas DHT and vehicle were largely ineffective. The additive actions of DHT and T on MSB are discussed in relation to different effects of these androgens on androgen receptors in the male hamster brain mating circuit.

## Introduction

Testosterone (T) has long been implicated in the maintenance of male sex behavior (MSB) [1]–[3]. In many mammals reduced androgen availability is associated with marked reductions in copulatory behavior. In some species androgens exert their effects directly on androgen receptors (ARs); in others, conversion of T by brain tissues may be an essential step [4]. The canonical view, derived from studies of rats, emphasizes conversion of T to estradiol (E_2_) by the enzyme aromatase as a key step in nervous system maintenance of MSB. Treatment with 5α-dihydrotestosterone (DHT) or E_2_ alone is not sufficient to maintain ejaculation in most castrated rats; combined treatment with both hormones maintains full copulatory ability, although less effectively than T treatment [5]. Physiologically relevant E_2_ plus DHT treatments that elevate brain androgen and estrogen receptor levels to those of castrated rats treated with T capsules that restore MSB, fail to reinstate copulation [6]. Nevertheless, both androgenic and estrogenic metabolites are thought to contribute to MSB in rats [7]–[8].

Because DHT, a non-aromatizable steroid, effectively maintains MSB in guinea pigs, rhesus monkeys, rabbits (reviewed in [9]), and some mouse strains [10], the generality of the aromatization hypothesis has been questioned. It remains unclear which of T's metabolites is critical for MSB in species other than rats [11].

In Syrian hamsters supraphysiological concentrations of E_2_ or DHT fail to restore MSB [12]–[13]. In a typical study only a cocktail of E_2_ and DHT that generates blood concentrations more than two orders of magnitude greater than those in intact males restores copulatory behavior, but even then not to the high levels displayed by intact males [12]–[13]; the biological relevance of such non-physiological treatments is questionable. Administration of the aromatase inhibitor fadrozole does not compromise any component of copulatory behavior in intact male Syrian hamsters, despite inhibiting brain formation of E_2_ from T [14].

Nuclear binding of T to the AR in brain and peripheral tissues is abolished when an excess of DHT is given simultaneously with T [15] and pretreatment with DHT suppresses binding of T [16]. In Syrian hamsters both DHT and T are ligands for ARs [17] and T treatment concurrent with injection of labeled DHT completely inhibits uptake of DHT [18]. Although it remains inconclusive whether separate ARs mediate effects of T and DHT [19]–[20], several investigators have suggested that these two androgens activate different target genes or that two kinds of androgen-response elements mediate differential AR transactivation by T and DHT [21]–[22]. In Syrian hamsters T and DHT differentially affect harderian gland mRNA [23], suggesting that the two hormones may activate different genes after binding to the AR. Based on these considerations we reasoned that if DHT and T occupy the same ARs, then treatment with a supraphysiological dose of DHT (8 times the endogenous concentration), that by itself does not support MSB, might inhibit the ability of otherwise effective T treatment to restore MSB. This presupposes that the DHT pretreatment saturates or substantially reduces the number of ARs available to T. Alternatively, if T and DHT act at different ARs, pretreatment with DHT may facilitate activation of MSB by low physiological dose T replacement, possibly by increasing total androgen availability or by increasing the number of brain AR immunoreactive cells [24].

## Materials and Methods

### Ethics Statement

All animal procedures were approved by the Animal Care and Use Committee at the University of California, Berkeley and were conducted in compliance with the NIH guide for the care and use of animals. Approval ID for this study is R084-0910C.

### Animals

Syrian hamsters (*Mesocricetus Auratus*; HsdHan: Aura; 10 weeks old) obtained from Harlan (Indianapolis, IN) were maintained on a 14L:10D photoperiod (14 h light/day, lights off at 1800 h PST). Tap water and Lab Diet Prolab 5P00 were continuously available.

Hamsters were singly housed at 23±1°C in polypropylene cages (48×25×21 cm) furnished with Tek-Fresh Lab Animal Bedding (Harlan Teklab, Madison, WI).

### Experimental Procedure

#### Screening for male sexual behavior

Adult male hamsters were 12 weeks old at the time they were screened in real time for MSB during the late portion of the light phase (∼1400–1700 h) with ovariectomized females rendered sexually receptive with standard estradiol plus progesterone treatments [25]. A Silastic capsule (Dow Corning, Midland, MI; 4 mm in length; ID 1.98 mm, OD 3.18 mm) filled with estradiol-17β (Sigma, St. Louis, MO) and sealed with silicone adhesive, was implanted s.c. on the day of ovariectomy; behavioral receptivity was induced by injecting females s.c. with 350 µg progesterone (Sigma) dissolved in peanut oil (2.5 mg/ml) 4 h prior to the sexual behavior testing sessions. Females were not utilized more frequently than once every four days.

The testing arena, kept in the room in which hamsters were housed, consisted of a clear Plexiglass box (41×21×21 cm) set above a slanted mirror to facilitate observation of intromissions and ejaculations. After 10 min, during which the male was acclimated to the apparatus, a sexually receptive female was introduced and MSB recorded. Males that ejaculated on two consecutive tests separated by at least a week were retained for the experiment. During the first screening test observations were limited to 15 min, which was adequate for each male to ejaculate at least once. On the second screening test hamsters were observed for 30 min, which permitted the emergence of the full suite of behaviors and provided baseline data for comparison with postoperative measures.

We recorded the number of mounts not accompanied by an intromission that preceded ejaculation, the number of intromissions that preceded ejaculation, latencies to the first mount, first intromission, and first ejaculation. Males that failed to display any of the behaviors during postoperative tests were assigned the maximum latency of 30 min for each behavior. After the preoperative tests, hamsters were assigned to groups that did not differ with respect to any of the recorded behaviors.

#### Surgical procedures

Hamsters were anesthetized with isoflurane vapors (Baxter Healthcare, Deerfield, IL) and castrated through a midline incision in the abdominal cavity. Incisions were closed with sterile sutures and wound clips (Mikron Auto Clip 9 mm, Becton Dickinson, Franklin Lakes, NJ). Hamsters were injected s.c. with the analgesic 5% buprenorphine (0.1 ml/animal), postoperatively (Hospira Inc., Lake Forest, IL).

#### Experimental Design

Sexually experienced males were re-tested 5 weeks after castration (wk 0) to verify the loss of MSB, characterized by absence of intromission and ejaculation behaviors. The day after loss of MSB was verified, hamsters were treated s.c. with three Silastic capsules (Dow Corning; 10 mm each in length; ID 1.98 mm, OD 3.18 mm) that were either empty (blank) or packed with powdered DHT. Capsule size was selected to generate supraphysiological concentrations of DHT likely to compete effectively with much lower doses of injected T, thereby providing a test of the ability of DHT to influence restoration of MSB by T. Beginning two weeks after capsule implantation, hamsters were injected daily s.c. with 0.1 ml of a 50/50% ethanol-distilled water solution that contained either 25 µg T or no hormone. This concentration was selected based on a previous study that established 15 µg T as a near threshold dose for restoration of MSB in hamsters [26]. The four treatment groups, each containing ten hamsters, received either Blank implants + Vehicle injections (control), Blank implants + T injections (T), DHT implants + Vehicle injections (DHT), or DHT implants + T injections (DHT + T). Sexual behavior was tested in the same manner as in the final pre-operative testing session, except tests were terminated if a hamster failed to intromit after 10 min. Tests occurred 4, 6, and 7 wks after implantation of capsules, corresponding to 2, 4, and 5 wks after the start of daily injections. Behavior tests commenced 5–7 h after the most recent injection.

T was administered in an aqueous rather than oil vehicle, because the former preparation is more effective for restoration of hamster MSB [26]. 50 µg/day T in oil failed to restore MSB [12], whereas 15 µg of aqueous T/day restored the ejaculatory reflex in 100% of castrated males [26]. The latter procedure was adopted in the present study on the assumption that T treatments that generate blood hormone concentrations orders of magnitude greater than those present physiologically [12] reduce the possibility of detecting interactions of T and DHT in the control of MSB. The physiologically relevant T replacement regimen of 25 µg/day [26] elevates blood T concentrations for several hours each day and more closely mimics the episodic secretion of T in intact males [27] than hormone replacement via implanted capsules or administration in oil vehicles (reviewed in [25]–[26]).

#### Blood and tissue analysis

All hamsters were bled one day after the wk 5 behavior test, 2 h after injection of hormone or vehicle. Blood was obtained from the retro-orbital sinus from hamsters lightly anesthetized with isoflurane vapors. Samples were centrifuged at 4°C for 20 min at 3000 rpm, and the serum collected and frozen at −80°C until assayed for concentrations of T, DHT and E_2_. Two subgroups were formed from the DHT hamsters depending on whether the individual ejaculated after replacement treatment (4 hamsters) or failed to ejaculate (6 hamsters generated 4 samples derived by pooling sera from 2 pairs of hamsters with insufficient blood volumes for individual assays). T concentrations from unpooled samples were determined for 4 hamsters from the DHT + T group, 4 hamsters from the T group and 5 hamsters from the control group. Unpooled samples from 6 hamsters from the DHT + T group, 6 from the T group, and 5 from control group were assayed for E_2_. The new groups created for blood hormone determinations did not differ with respect to sexual behavior within groups; i.e., T-injected hamsters assayed for T did not differ from T-injected hamsters assayed for E_2_.

Approximately 48 h after the final behavior test hamsters were euthanized by placement in a carbon dioxide-filled chamber; the seminal vesicles, ventral prostate, and penis were removed, and dry weights recorded (±0.01 mg).

#### Hormone radioimmunoassay

Hormone concentrations were measured with commercially available radioimmunoassay kits (Diagnostic Systems Laboratory, Inc, Webster, TX) previously validated for use with non-extracted Syrian hamster serum, which included a description of parallelism upon serial dilution, and a recovery of 94% [28]. The T and DHT assays (DSL 4000 and 9600, respectively), were validated for Syrian hamster serum by Faruzzi et al. [29].

Samples for T were assayed in duplicate (DSL-4000), whereas samples for E_2_ were determined in singleton (DS-43100), owing to the larger sample volume requirement of that assay. Serum samples underwent an oxidation/extraction procedure prior to the DHT assay (DSL-9600). The intra-assay correlations of variation for T, E_2_, and DHT were 8.9, 2.0, and 3.1%, respectively, and the minimum detection limits were 0.08 ng for T, 0.01 ng for E_2_, and 0.004 ng/ml for DHT, respectively. Samples were run in a single assay for each hormone. Cross-reactivity with T was 0.02% in the DHT assay after extraction; in the T assay cross-reactivity with DHT was 5.8%, as reported by the manufacturer.

#### Statistical analyses

Differences between pre- and postoperative sex behaviors were assessed with paired t-tests. Mixed ANOVAs compared postoperative, hormone-treated groups across time. Differences between groups at specific time points or for post-mortem tissue weight analyses were assessed with single factor ANOVA followed by pairwise comparisons using Tukey's HSD test. If an omnibus ANOVA could not be carried out due to too few data points in some groups, those groups were excluded from analyses; e.g., at wk 5 no hamsters from the vehicle group and only one from the DHT group ejaculated, which does not allow for statistical comparison; thus, these groups were eliminated from the analysis. Comparisons of proportions of hamsters in each group displaying a behavior were calculated with the Fisher-Freeman-Halton Exact Test. The Statview program (Statview 5; SAS Institute, Cary, NC) was used for all ANOVA and t-tests. The Fisher-Freeman-Halton Exact test was computed with the Statsdirect program (Statsdirect 2.7.7; Altrincham, UK). Observed differences were considered significant if p<0.05 and are reported as such.

## Results

There were no differences between groups in the numbers of hamsters displaying mounts, intromissions, or ejaculations during pre-operative testing. All hamsters failed to intromit or ejaculate 5 weeks after castration (wk 0 test).

### Restoration of male sexual behavior after hormone treatments

#### Ejaculatory Behavior

**Figure 1:**
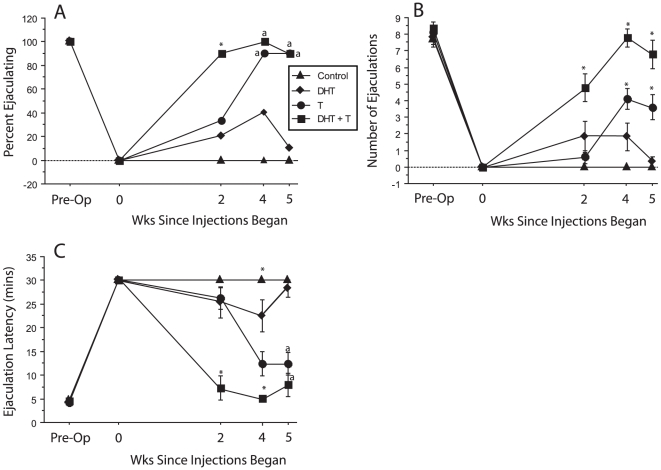
DHT and T synergize to accelerate restoration of ejaculatory behavior. Percent of males displaying the ejaculatory reflex (A), number of ejaculations per 30 min test (B) and ejaculation latencies on the first ejaculatory series (C), during post-castration tests 2–5 wks after the onset of hormone or vehicle injections. * Significantly different from all other groups. “a” significantly different from the control group (p<0.05).

All hamsters were treated with DHT or empty capsules beginning 2 wks prior to the start of the injection regimen; capsules remained *in situ* for the duration of the experiment. None of the control males treated with blank capsules and subsequently injected with vehicle displayed the ejaculatory reflex during any of the post-operative tests. After 2 wks of hormone injection, however, ejaculatory behavior was displayed by 90% of the DHT + T group, compared to 30% of the T group (p<0.05) and 20% of the DHT males (Fig. 1A, wk 2, p<0.05). Combined treatment with both hormones substantially accelerated reinstatement of ejaculatory behavior. After 5 wks of treatment (wk 5, Fig. 1A), 90% of males in both the DHT + T and T groups ejaculated, compared to 10% of the DHT males (p<0.05). With continued treatment T was as effective as DHT + T in restoring the ejaculatory reflex in 90% of males. DHT and control males did not differ significantly at any time point.

The number of ejaculations per test differed significantly across time (F_3,35_ = 61.1, p<0.05) and a significant group × time interaction was detected (p<0.05). At wk 2, the DHT + T group ejaculated significantly more often than each of the other 3 groups (Fig. 1B). The time between wks 2 and 4 accounted for this interaction (p<0.05). By wks 4 and 5, all groups differed significantly from each other except for the DHT and control hamster comparison (Fig. 1B). The number of ejaculations by males treated with both hormones did not differ from preoperative values, indicative of full restoration of MSB, whereas males treated with just T ejaculated significantly less often than during preoperative testing.

Hormone treatment significantly affected ejaculation latencies (ELs) across groups (F_3,36_ = 51.5, p<0.05); ELs were significantly shorter for DHT + T hamsters than for all other groups at wks 2 and 4 (Fig. 1C); by wk 5, ELs were equal in the DHT + T and T groups. Only treatment with DHT + T restored ELs to preoperative values. At wks 4 and 5, ELs of the T group were significantly shorter than those of the control and DHT hamsters. Again, there was a significant interaction between the groups and time (p<0.05), with the changes between wks 2 and 4 accounting for this effect (p<0.05).

#### Intromission Behavior

**Figure 2:**
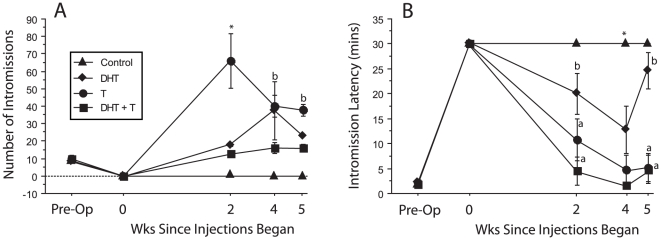
DHT + T treatment reduces the number of intromissions preceding ejaculation. Number of intromissions (A) and intromission latencies (B), preceding the first ejaculation. * Significantly different from all other groups “a” significantly different from control group). “b” significantly different from DHT + T group.

DHT + T hamsters displayed significantly fewer intromissions prior to ejaculation than T hamsters at wks 2, 4 and 5 (Fig. 2A). Values for DHT + T hamsters at wk 5 did not differ significantly from preoperative values; T hamsters, in contrast, had substantially more intromissions prior to ejaculation at wk 5 than preoperatively (p<0.05).

Hormone treatment significantly altered intromission latencies (ILs) across time (F_3,35_ = 40.2, p<0.05); hamsters that failed to intromit during all postoperative behavior tests were assigned the 30 min default value. There was no interaction between group and time (p>0.05). DHT + T and T males maintained preoperative-like ILs at wks 2, 4, and 5, that were significantly shorter than those of the control and DHT groups. By wk 5, DHT was no more effective than vehicle at reducing ILs.

#### Mounting Behavior

**Figure 3:**
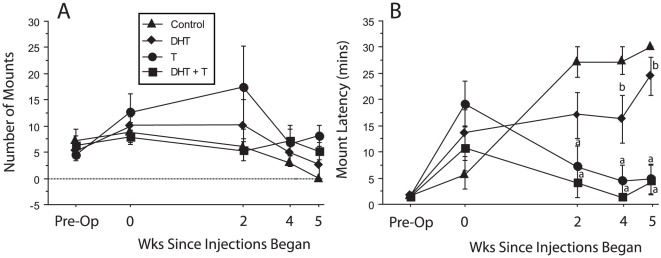
T and DHT + T treatments do not affect number of mounts but reduce mount latencies. Number of mounts (A) and mount latencies (B) preceding the first ejaculation. “a” significantly different from control group “b” significantly different from DHT + T group. Mount numbers did not differ significantly between groups over time.

After non-ejaculators were excluded from the data analysis, the number of mounts that preceded the first ejaculation did not differ among groups (F_2,11_ = 1.1, p>0.05; Fig. 3A). In addition, there was no significant interaction between the time and group factors (p>0.05). Mount latencies (MLs) differed significantly as a function of hormone treatment (F_3,35_ = 33.2, p<0.05). T and DHT + T groups had significantly shorter MLs than did the control and DHT groups at wks 2, 4 and 5; DHT and control groups did not differ significantly (p>0.05). The T and DHT + T groups maintained MLs during all postoperative tests that did not differ significantly from preoperative values.

#### Ventral Prostate, Seminal Vesicle and Penis Weights

**Figure 4:**
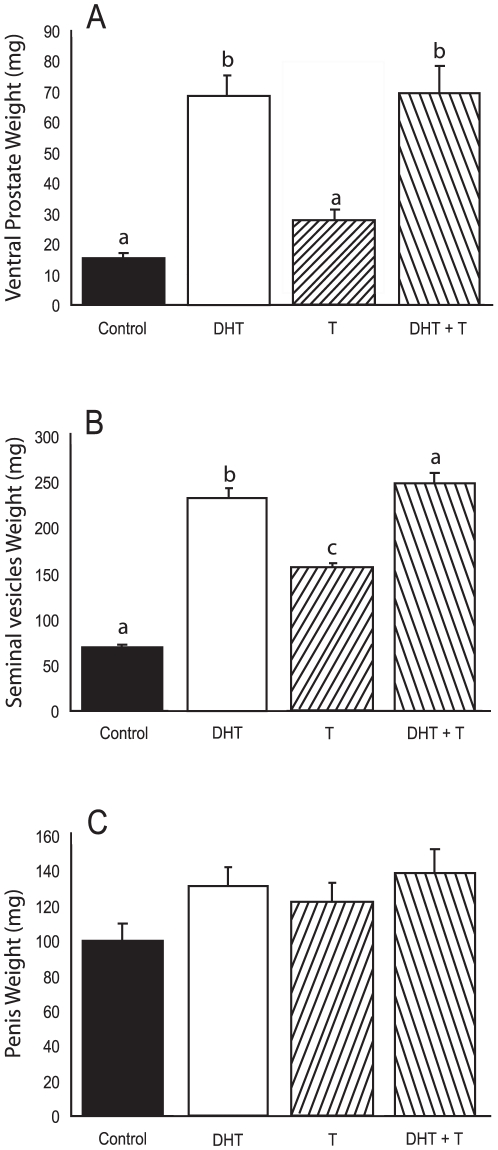
DHT alone or in combination with T increases prostate and seminal vesicle but not penis weights. Ventral prostate (A) seminal vesicle (B) and penis weights (C) (mg). Bars designated with different letters differ significantly from one another DHT increased prostate and seminal vesicle weights.

Differential hormone treatment resulted in significantly different ventral prostate and seminal vesicle weights (F_3,35_ = 24.0, F_3,35_ = 97.9, respectively, p<0.05). Administration of DHT, either alone or in combination with T, resulted in significantly increased ventral prostate and seminal vesicle weights compared to those of other groups (Fig. 4A, 4B; respectively) but the DHT + T and DHT groups did not differ. T administration alone did not increase ventral prostate weight, but did significantly increase seminal vesicle weight, compared to values of vehicle-treated controls. Penile weights did not differ significantly based on a single factor ANOVA analysis (F_3,35_ = 2.4, p = 0.11; Fig. 4C).

#### Blood hormone concentrations

**Figure 5:**
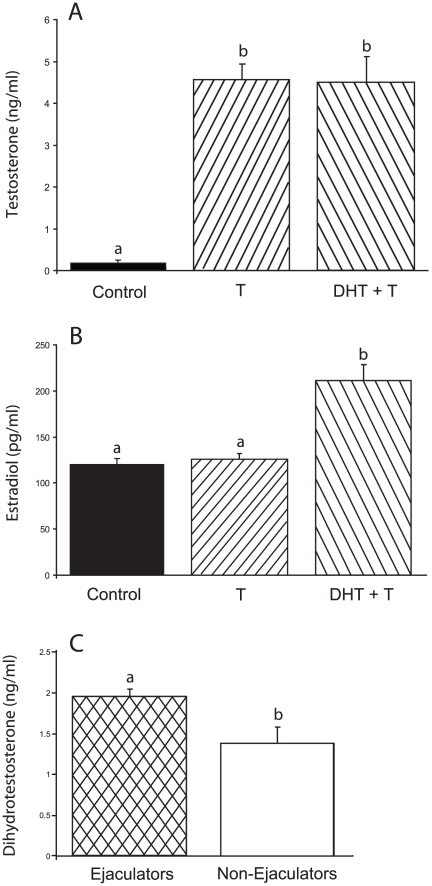
T and DHT + T elevate blood T concentrations; DHT + T increases E_2_ concentrations. Higher DHT concentrations are associated with increased ejaculatory behavior. (A) Testosterone, (B) estradiol, and (C) dihydrotestosterone concentrations. Bars designated with different letters differ significantly from one another N = 4–5 for each of the groups in panel A and 5–6 in panel B. C. N = 4 and 6 for hamsters that ejaculated or were non-ejaculators, respectively.

T and DHT + T injections each significantly elevated T concentrations compared to the vehicle-injected control group; the T and DHT + T groups did not differ from each other (Fig. 5A).

Both T and control groups, had significantly lower E_2_ concentrations than the DHT + T group. DHT males that had ejaculated on one or more of the tests (n = 4) had significantly higher DHT concentrations than those that did not ejaculate (p<0.05; Fig. 5C).

## Discussion

Pretreatment with DHT, although itself ineffective for restoring MSB, combined with T to restore copulatory behavior to values generated by intact males. Any competition of these steroids for available ARs did not negatively affect the display of MSB. On the contrary, after two weeks, the combined treatment was far more effective than T alone in restoring MSB. This is the first such demonstration in any mammal, except for an unpublished study by Arteaga-Silva et al. ([13] p. 419), who noted that “…simultaneous treatment with T and DHT is more effective than T alone to restore MSB in castrated hamsters”. The more rapid restoration of MSB by DHT + T treatment may involve metabolism of DHT to 3β–diol, a hormone effective in restoring MSB in rats via actions on ER-β [30]. This metabolite restored sexual behavior in ∼half of the rats tested by Morali et al. [31], but was only fully effective when co-administered with DHT. Our observation that E_2_ concentrations were twice as high in the DHT + T than in T-treated or control hamsters does not preclude the involvement of estrogens in activation of MSB. Both DHT and its metabolite, 3β-diol, can upregulate aromatase activity, which may account for these differences [32]; there is, however, little definitive evidence for the regulation of the ejaculatory reflex by E_2_ in Syrian hamsters. Nor is it known whether or not brain ER-β activation affects MSB in Syrian hamsters. Circulating E_2_ concentrations in the present study were much lower than those necessary to synergize with DHT to partially restore MSB [12].

The more rapid restoration of MSB by DHT + T treatment suggests an additive effect of T and DHT on the hamster brain mating circuit [33] or more effective restoration of penile spines by DHT than T [13], but behavioral potency of steroids did not correlate with stimulation of penile growth [13].

The long-term maintenance of localized nuclear ARs by DHT pretreatment also may contribute to decreased latencies for restoration of MSB by T in castrated males. Two months after castration, steady-state brain AR mRNA is decreased; administration of DHT restores values in the bed nucleus of the stria terminalis and medial preoptic area of rats to within the intact range [34]–[35]. DHT may accelerate resumption of MSB in response to T treatment through rapid reinstatement of AR mRNA, or by inducing higher levels of AR protein [30], thereby yielding more binding sites for the T ligand. Blood DHT concentrations generated by the implants were an order of magnitude higher than those reported for intact hamsters [12] and well above concentrations generated by 5 mm implants that “maintain androgen receptor immunoreactivity exclusively within the neuronal cell nucleus” [36], which suggests that our dose of DHT was sufficient. AR occupancy may be necessary but is not a sufficient condition for activation of MSB in Syrian hamsters [37].

The complete pattern of MSB was restored in 90% of hamsters by daily injection of a physiological dose of 25 µg T, but the number of ejaculations was reduced well below preoperative values and the number of intromissions preceding ejaculation increased substantially, suggesting that this was a suboptimal replacement regimen. The T dose employed in the present study was much lower than those typically administered in replacement protocols (500–1000 µg); it generated blood T concentrations twice the normal physiological value of 2 ng/ml for a short time, with decreases to below 0.9 ng/ml no later than 7 h after injection [26]. The amount of T present for most of the interval between injections is unlikely to compete effectively with DHT for ARs, given chronic DHT concentrations of ∼1.3 to 2.0 ng/ml produced by the Silastic capsule implants. Approximately 10 times higher concentrations of T are required to produce the AR transcription effects of DHT [38].

DHT restored MSB in a small number of hamsters, but less robustly than in hamsters treated with T or DHT + T, as evidenced by increased latencies to intromit and ejaculate; longer ejaculation latencies in DHT than T-treated males were previously reported [39]. When analyses were restricted to hamsters that ejaculated, latencies were similar in DHT and T-treated hamsters. This effect was transient, however, as only one of the DHT hamsters that ejaculated at wk 4 also ejaculated at wk 5. It is unknown why DHT is much less effective than T for restoring MSB in Syrian hamsters or whether 5-α reduction of T to DHT is implicated in the control of MSB in intact males.

T treatment increased seminal vesicle but not ventral prostate weights. Higher doses of T restore both tissues to preoperative values [40]. In the presence of the low dose of T administered in the present study sensitivity of the seminal vesicles to T is greater than that of the ventral prostate. DHT was effective at increasing both seminal vesicle and ventral prostate weights to the same extent, with or without T supplementation, which confirms many earlier studies that DHT is more potent than T in maintaining peripheral androgen-responsive tissues. The seminal vesicle and prostate weights, were, however, lower than those of intact males in a previous study from our laboratory [25], suggesting that treatment duration or steroid doses were suboptimal for these tissues.

To the extent that DHT competes with T for AR binding sites, it does not interfere with sex behavior-promoting actions of T. Rather, MSB is facilitated by co-administration of the two hormones, which may attest to their differing interactions with the AR, to unexplored effects of DHT metabolites, or to the greater duration or amount of androgen exposure in hamsters treated with both hormones.
